# Blocking and Binding Folate Receptor Alpha Autoantibodies Identify Novel Autism Spectrum Disorder Subgroups

**DOI:** 10.3389/fnins.2016.00080

**Published:** 2016-03-09

**Authors:** Richard E. Frye, Leanna Delhey, John Slattery, Marie Tippett, Rebecca Wynne, Shannon Rose, Stephen G. Kahler, Sirish C. Bennuri, Stepan Melnyk, Jeffrey M. Sequeira, Edward Quadros

**Affiliations:** ^1^Department of Pediatrics, Arkansas Children's Hospital Research Institute, University of Arkansas for Medical SciencesLittle Rock, AR, USA; ^2^Department of Medicine, State University of New York–Downstate Medical CenterBrooklyn, NY, USA

**Keywords:** folate receptor autoantibody, autism spectrum disorders, folinic acid, redox metabolism, glutathione

## Abstract

Folate receptor α (FRα) autoantibodies (FRAAs) are prevalent in autism spectrum disorder (ASD). They disrupt the transportation of folate across the blood-brain barrier by binding to the FRα. Children with ASD and FRAAs have been reported to respond well to treatment with a form of folate known as folinic acid, suggesting that they may be an important ASD subgroup to identify and treat. There has been no investigation of whether they manifest unique behavioral and physiological characteristics. Thus, in this study we measured both blocking and binding FRAAs, physiological measurements including indices of redox and methylation metabolism and inflammation as well as serum folate and B12 concentrations and measurements of development and behavior in 94 children with ASD. Children positive for the binding FRAA were found to have higher serum B12 levels as compared to those negative for binding FRAAs while children positive for the blocking FRAA were found to have relatively better redox metabolism and inflammation markers as compared to those negative for blocking FRAAs. In addition, ASD children positive for the blocking FRAA demonstrated better communication on the Vineland Adaptive Behavior Scale, stereotyped behavior on the Aberrant Behavioral Checklist and mannerisms on the Social Responsiveness Scale. This study suggests that FRAAs are associated with specific physiological and behavioral characteristics in children with ASD and provides support for the notion that these biomarkers may be useful for subgrouping children with ASD, especially with respect to targeted treatments.

## Introduction

Autism spectrum disorder (ASD) is a devastating neurodevelopmental disorder with life-long consequences that affects young children during critical times in their development. The Center for Disease Control estimates that 1 in 68 individuals in the United States (~1–2%) are affected by an ASD (Developmental Disabilities Monitoring Network Surveillance Year 2010 Principal Investigators; Centers for Disease Control and Prevention (CDC), 2014). Despite this alarming prevalence, the etiology of ASD is still poorly understood with several studies suggesting that both environmental and genetic components may contribute equally to the etiology of ASD (Hallmayer et al., 2011; Sandin et al., 2014).

Recent research has suggested that several physiological abnormalities such as dysfunctional mitochondrial and redox metabolism and immune abnormalities, alone, or in combination, are related to the underlying aberrant biological processes that results in ASD (Rossignol and Frye, 2012b). Folate is related to many of the physiological systems found to be abnormal in ASD (Frye and James, 2014) and has been shown to be protective against the development of ASD when supplemented in adequate amounts during or before pregnancy (Schmidt et al., 2012; Suren et al., 2013). Folate is a water-soluble B vitamin that is essential for numerous metabolic reactions and normal neurodevelopment (Greenblatt et al., 1994; Black, 2008).

In some individuals with ASD, the primary mechanism that transports folate across the blood-brain barrier may be compromised. The folate receptor alpha (FRα) along with the energy dependent endocytosis transports folate, attached to the FRα, from the apical to the basolateral side of the choroid plexus endothelium against a concentration gradient. Active transport of folate is necessary because the folate concentration in the central nervous system is several times higher than the folate concentration in the blood (Frye et al., 2013b).

A new neurometabolic disorder called cerebral folate deficiency (CFD) was described about a decade ago. CFD is characterized by abnormally low folate concentrations in the cerebrospinal fluid despite normal folate concentration in the serum (Ramaekers et al., 2002). CFD is associated with autoantibodies to the FRα which impair its function (Ramaekers et al., 2005). Serum titers of FRα autoantibodies (FRAAs) have been correlated with cerebrospinal fluid (CSF) folate concentrations in independent studies (Ramaekers et al., 2005; Frye et al., 2013b). Mitochondrial disorders can cause CFD because of the lack of energy for the active transportation of folate across the blood-brain barrier (Ramaekers et al., 2007b; Garcia-Cazorla et al., 2008; Frye and Naviaux, 2011). Early case-series of children with CFD described many with ASD features (Ramaekers and Blau, 2004; Ramaekers et al., 2005).

Recently, Frye et al. (2013b) measured blocking and binding FRAAs in 93 children with ASD using the assay developed by Dr Quadros (Ramaekers et al., 2005; Molloy et al., 2009). Overall, 60 and 44% were positive for the blocking and binding FRAAs, respectively. The high prevalence of FRAAs in ASD children was verified in a study from Belgium which demonstrated that 47% of children with ASD were positive for the blocking FRAA as compared to 3.3% of developmentally delayed non-ASD controls (Ramaekers et al., 2013b). These prevalence rates for blocking FRAAs are clearly higher than prevalence rates in healthy populations which vary from 10 to 15% (Frye et al., 2013b).

The importance of the FRα is related to the targeted treatment which can bypass the FRα when it is blocked and/or dysfunctional. Folinic acid can cross the blood-brain barrier using the reduced folate carrier (RFC) when the FRα is blocked by FRAAs or non-functional due to mitochondrial dysfunction and/or genetic mutations. The RFC has a lower affinity for folate than the FRα, so a high-dose of folinic acid is required for treatment. Case-reports (Moretti et al., 2005) and series (Ramaekers et al., 2005, 2007a) have described neurological, behavioral and cognitive improvements in children with CFD and ASD with high-dose folinic acid (0.5–2 mg/kg/day), including complete recovery of ASD symptom in some and substantial improvements in communication in many (Ramaekers et al., 2005, 2007a).

Frye et al. (2013b) treated 44 children positive for at least one FRAA with high-dose folinic acid in an open-label fashion. After 4 months of treatment, significant improvements in verbal communication, receptive and expressive language and stereotypical behavior were noted in the treated children as compared to a wait list control group who were also FRAA positive. About two-thirds (66%) of treated children showed some improvement in language, verbal communication and stereotyped behavior, with one-third (~33%) demonstrating moderate or much improvement in these areas.

Although, this promising evidence suggests that FRAAs are biomarkers that can identify an important subset of children with ASD who may respond to a specific treatment, an investigation into whether the subgroup of ASD children with FRAA have particular characteristics that can distinguish them from others with ASD, has not been conducted. Thus, in this study we examine a sample of children with ASD to determine the correspondence between FRAA status and behavior and developmental characteristics. Since the folate pathway is closely connected with methylation and redox regulation pathways (Frye and James, 2014), markers of glutathione and methylation metabolism are also examined. Since antibody production can be associated with immune activation and increases in inflammation can be associated with redox and methylation abnormalities (Rose et al., 2012; Rossignol and Frye, 2014), a marker of inflammation is also examined.

Since there are two different FRAAs, blocking and blinding, our analysis determines whether the characteristics investigated are an effect of blocking or binding FRAAs independently of one another. Since some individuals will have both blocking and binding FRAAs, a general linear model is used to investigate these independent effects. This will be the first time a difference between these two FRAAs has been investigated. Such information can not only help better identify children with ASD who are positive for FRAAs but can help us understand the consequence and significance of each FRAA in ASD.

## Materials and methods

The data from the 94 children with ASD were obtained from two research protocols approved by the Institutional Review Board at the University of Arkansas for Medical Science (Little Rock, AR). Written informed consent was obtained from parents of participants and assent was waived.

A diagnosis of ASD, required for study entry, was defined by one of the following: (i) a gold-standard diagnostic instrument such as the Autism Diagnostic Observation Schedule and/or Autism Diagnostic Interview-Revised (ADI-R); (ii) the state of Arkansas diagnostic standard, defined as agreement of a physician, psychologist and speech therapist; and/or (iii) Diagnostic and Statistical Manual for Mental Disorders diagnosis by a physician along with standardized validated questionnaires and diagnosis confirmation by the Principal Investigator (REF). Reconfirmation of the diagnosis using the ADI-R by an independent research reliable rater was requested for a portion of participants to confirm that the criteria used for including the participants was equivalent to other high-quality studies. Excluded from the study were children on antipsychotic medications as well as children with well-defined genetic syndromes.

### Folate autoantibody assay

About 1 ml of serum was analyzed at the State University of New York, Downstate (Brooklyn, NY) for blocking and binding FRAAs using the assay previously described (Ramaekers et al., 2005; Molloy et al., 2009). Blocking FRAAs are expressed as pmoles of folic acid blocked from binding to FRα per ml of serum, and binding FRAAs are expressed as pmoles of IgG antibody per ml of serum.

### Redox, methylation, immune and vitamin biomarkers

Redox and methylation potential was measured by the total and free reduced-to-oxidized glutathione redox ratio (tGSH/GSSG and fGSH/GSSG) and the S-adenosylmethionine to S-adenosylhomocysteine ratio (SAM/SAH), respectively. 3-Chlorotyrosine (CT), a measure of myeloperoxidase activity, was used as a marker of immune system activation. Fasting blood (4 ml) was collected into an EDTA-Vacutainer tube, chilled on ice and centrifuged at 1500 × g for 15 min at 4°C. Plasma was stored at −80°C and analyzed by HPLC with electrochemical detection as previously described (Melnyk et al., 1999) within 2 weeks of collection. Total plasma folate and vitamin B12 were measured using MP Diagnostics SimulTRAC-SNB Radioassay Kit (Cat# 06B264806).

### Cognitive and behavioral assessments

The Preschool Language Scale-4 (PLS) and two versions of the Clinical Evaluations of Language Fundamentals (CELF) were used to assess language ability. The CELF is one of the only standardized, well-validated language assessment instruments that spans the age range of most participants (using both CELF-preschool-2 and CELF-4; Semel et al., 2003; Wiig et al., 2004). It assesses a wide range of language skills that are only partially measured by other language tests, including high-level language skills that are abnormal in individuals with ASD, such as language pragmatics (Condouris et al., 2003) and has been used in several studies focusing on core language deficits in ASD (Verly et al., 2014; Edgar et al., 2015). The PLS is also a standardized, well-validated language assessment instrument that can measure subtle changes in language in children with poor language abilities (Zimmerman et al., 2002; Volden et al., 2011). Both instruments provide a standardized core language score which was used as the index of language ability. For each participant the most ability appropriate instrument was used in order to prevent floor and ceiling effects.

Adaptive behavior was assessed using the Vineland Adaptive Behavior Scales, 2nd Edition, Interview Edition, Survey Form (VABS), an instrument that has demonstrated good reliability and validity (Sparrow et al., 2005). Standardized scores for summary scales were examined: communication, daily living skills, social skills, motor skills and adaptive behavior composite.

The Aberrant Behavior Checklist (ABC) was designed to measure disruptive behaviors in individuals with developmental disabilities (Aman et al., 1985). The ABC has been shown to have convergent and divergent validity in ASD (Kaat et al., 2014) and has been used in multiple autism clinical trials (Frye et al., 2015).

The Social Responsiveness Scale (SRS) measures the severity of social skill deficits (Constantino, 2002). It has been validated and shown to be reliable and to have good correspondence to the gold-standard ADI-R, while being more time efficient and cost effective (Murray et al., 2011).

### Statistical analysis

The “glm” procedure of SAS 9.1 (SAS Institute Inc., Cary, NC) was used with a two-tailed alpha of 0.05. FRAA status was dichotomized into positive or negative for both blocking and/or binding FRAAs. These variables were entered into a general linear model to determine if differences in the dependent variable were related to blocking and/or binding FRAA status. In this sense, the analysis examined the independent effects of blocking and bindings FRAAs on the dependent variable while controlling for the effect of each FRAA. Because of the limited sample size, the interaction of the two FRAAs was not examined. Planned orthogonal contrasts were used to determine whether the blocking and binding FRAAs demonstrated significant differences in the dependent variable. Since ASD symptoms are often associated with developmental level, the VABS Adaptive Behavior Composite was used as a covariate in the analysis that examined behavior and developmental indices.

## Results

### Participants

Basic participant characteristics are outlined in Table 1 stratified across FRAA status. The average age and gender did not differ across the FRAA groups. Race and ethnicity was not different across FRAA groups. Overall, 84% were Caucasian, 7% African American, 5% Asian and 3% mixed race and 95% were non-Hispanic.

**Table 1:** **Demographic and clinical characteristics by folate receptor alpha autoantibody groups**.

**Variable**	**FRAA Negative (*n* = 40)**	**FRAA Blocking Positive (*n* = 16)**	**FRAA Binding Positive (*n* = 48)**
Age, years months	7 years 0 months (3 years 4 months)	6 years 5 months (3 years 0 months)	7 years 4 months (3 years 2 months)
Males, N (%)	34 (85%)	15 (94%)	39 (81%)
Vineland adaptive behavior composite,	64.9 (11.7)	66.7 (10.8)	63.2 (10.4)
Only blocking autoantibody positive, N (%)		6 (38%)	
Only binding autoantibody positive, N (%)			38 (79%)
Both folate autoantibodies positive, N (%)		10 (63%)	10 (21%)
Blocking titer (pmol/ml),		0.52 (0.36)	0.54 (0.44)
Binding titer (pmol/ml),		1.03 (0.81)	0.85 (0.66)
Glutathione redox ratio, total	30.72 (1.09)	29.67 (1.07)	34.92 (2.06)
Glutathione redox ratio, free	9.06 (1.83)	9.82 (1.82)	8.75 (1.62)
Methylation SAM/SAH Ratio	2.65 (0.48)	2.67 (0.42)	2.60 (0.46)
3-Chlorotyrosine	37.7 (7.6)	33.4 (6.6)	37.1 (7.7)
Folate (ng/ml) [Normal 5-21],	17.7 (4.3)	18.9 (5.3)	18.0 (4.1)
B12 (pg/ml) [Normal 200-900],	828.3 (451.3)	1426.0 (1589.8)	1383.0 (1495.4)
**LANGUAGE TESTING, N (%)**			
Preschool language scales	12 (30%)	4 (25%)	17 (35%)
Clinical evaluation of language fundamentals 2	14 (35%)	6 (38%)	10 (21%)
Clinical evaluation of language fundamentals 4	13 (33%)	6 (38%)	21 (44%)
**DIAGNOSTIC DOCUMENTATION, N(%)**			
Autism diagnostic observation schedule	20 (50%)	8 (50%)	22 (46%)
3 Practitioner agreement	30 (75%)	10 (63%)	31 (65%)
Single practitioner with standardized questionnaires	5 (13%)	5 (31%)	12 (25%)
**AUTISM DIAGNOSTIC INTERVIEW-REVISED**			
Participated in confirmation testing, N(%)	27 (68%)	11 (69%)	37 (77%)
Social interaction score,	21.41 (5.44)	22.18 (4.40)	22.76 (4.96)
Communication score: verbal,	16.64 (4.40)	18.88 (1.46)	19.43 (3.89)
Communication score: non-verbal,	12.69 (2.36)	12.33 (2.08)	13.07 (1.59)
Restricted and repetitive play score,	5.85 (1.83)	5.82 (2.71)	5.84 (2.39)
Summary score,	4.41 (0.93)	4.64 (0.50)	4.41 (0.93)
**MEDICATIONS (CONCURRENT TREATMENTS), N (%)**			
Melatonin	17 (43%)	1 (6%)	10 (21%)
Allergy/Asthma medications	13 (33%)	4 (25%)	10 (21%)
Gastrointestinal medications	15 (38%)	2 (13%)	9 (19%)
Alpha-adrenergic agonists	7 (18%)	1 (6%)	10 (21%)
Stimulant	6 (15%)	0 (0%)	8 (17%)
Antiepileptic medication	4 (10%)	0 (0%)	6 (13%)
Antimicrobial medications	5 (13%)	1 (6%)	4 (8%)
Selective serotonin reuptake inhibitors	0 (0%)	1 (6%)	6 (13%)
Other psychotropic medications	4 (10%)	0 (0%)	2 (4%)
Immunomodulatory medications	2 (5%)	1 (6%)	2 (4%)
Antipsychotic	2 (5%)	0 (0%)	0 (0%)
Beta blocker	1 (3%)	0 (0%)	0 (0%)
**SUPPLEMENTS (CONCURRENT TREATMENTS), N (%)**			
Multivitamin	13 (33%)	6 (38%)	12 (25%)
Minerals	8 (20%)	3 (19%)	11 (23%)
Fatty acids	7 (18%)	4 (25%)	10 (21%)
Folate	8 (20%)	0 (0%)	7 (15%)
Vitamin B-12	2 (5%)	2 (13%)	12 (25%)
Carnitine	6 (15%)	3 (19%)	7 (15%)
Other antioxidants	4 (10%)	1 (6%)	8 (17%)
Other vitamins	7 (18%)	1 (6%)	5 (10%)
Other B vitamins	3 (8%)	3 (19%)	8 (17%)
CoEnzyme Q10	4 (10%)	1 (6%)	5 (10%)
Other supplements	1 (3%)	0 (0%)	3 (6%)
Amino acids	1 (3%)	1 (6%)	2 (4%)
Thyroid supplements	1 (3%)	0 (0%)	1 (2%)
**COMORBID MEDICAL CONDITIONS, N (%)**			
Allergic disorders	18 (45%)	6 (38%)	19 (40%)
Gastrointestinal disorders	18 (45%)	6 (38%)	17 (35%)
Neurological disorders	9 (23%)	5 (31%)	15 (31%)
Copy number variants	7 (18%)	4 (25%)	14 (29%)
Psychiatric disorders	5 (13%)	3 (19%)	14 (29%)
Immune abnormality	8 (20%)	1 (6%)	10 (21%)

*Mean values with standard deviation in parenthesis*.

Participants were recruited from our research registry (35%), autism clinic (27%), word-of-mouth (15%), physician referrals (13%), and community advertisement and social media (11%). All participants evaluated by an independent research reliable rater exceeded the threshold for the autism diagnosis.

Overall, immune and neurological abnormalities did not differ across FRAA status. Of the children with immune disorders, 79% had recurrent infections, 35% were diagnosed with immune disorder not otherwise specified, 32% had eczema, 21% had Pediatric Acute-onset Neuropsychiatric Syndrome, 11% had hypogammaglobulinemia and 5% had complement deficiency. Of the children with neurological abnormalities, 42% had epilepsy, 27% had migraines, 23% had abnormal electroencephalograms without seizures, 20% had visual problems, 15% had macrocephaly, 8% had Chiari malformation, 8% had non-specific magnetic resonance imaging abnormalities of the brain, and 4% had microcephaly.

### Folate receptor alpha autoantibodies

Fifty seven percent of the participants were positive for either the blocking or binding FRAAs, with 17% positive for blocking FRAA and 51% positive for the binding FRAA; 11% were positive for both FRAAs. There was no significant difference in age across participants who were FRAA blocking positive vs. negative or FRAA binding positive vs. negative.

### Folate and B12

**Figure 1:**
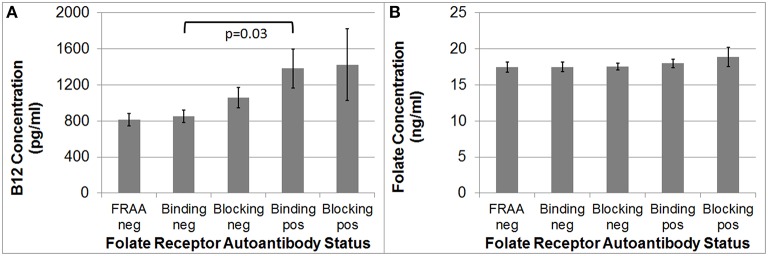
**(A)** Children with Autism Spectrum Disorder who are positive for the binding Folate Receptor Alpha Autoantibody have higher serum B12 concentrations than children negative for the binding Folate Receptor Alpha Autoantibody; **(B)** Serum Folate concentration does not differ across Folate Receptor Alpha Autoantibody status in children with Autism Spectrum Disorder.

B_12_ [*F*_(1, 92)_ = 4.35, *p* = 0.03], but not folate, was significantly higher in the binding FRAA positive participants as compared to binding FRAA negative participants (See Figure 1). Participants positive for the blocking FRAA were not, as a group, significantly different than those negative for the blocking FRAA with respect to B_12_ or folate. Neither, B_12_ nor folate was significantly different in those positive for blocking FRAAs vs. those positive for the binding FRAAs.

### Redox, methylation and inflammation biomarkers

**Figure 2:**
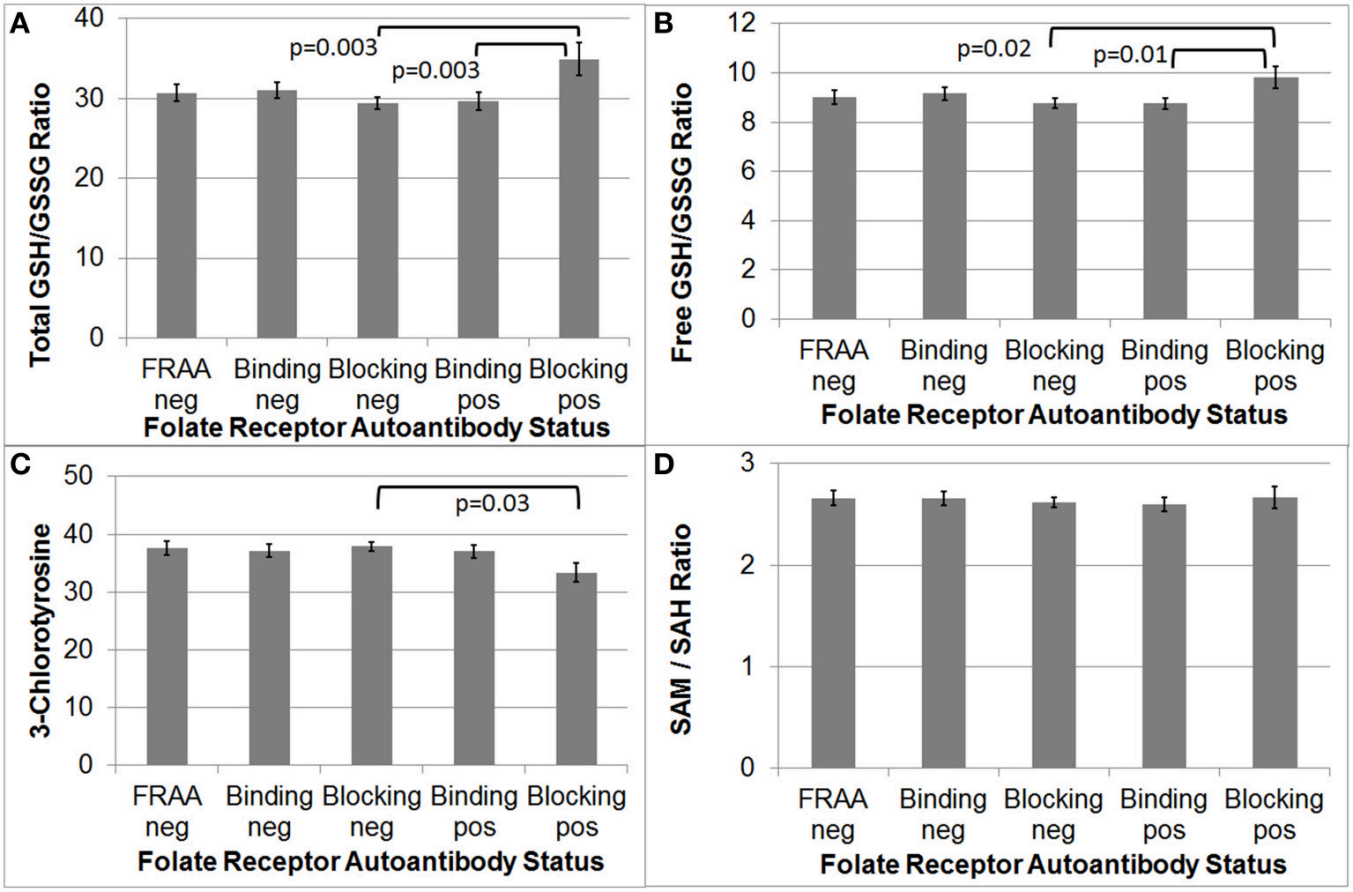
**Children with Autism Spectrum Disorder who are positive for the blocking Folate Receptor Alpha Autoantibody have more favorable total and free glutathione redox ratios (A,B) and marker of inflammation (C) as compared to those negative for the blocking FRAA. (D)** Methylation metabolism was not different across Folate Receptor Alpha Autoantibody status.

As depicted in Figure 2, total and free GSH/GSSG was significantly higher in participants positive for the blocking FRAA as compared to those participants negative for the blocking FRAA [*F*_(1, 92)_ = 9.52, *p* = 0.003 and *F*_(1, 92)_ = 5.35, *p* = 0.02, respectively]. Total and free GSH/GSSG were significantly higher in participants positive for blocking FRAAs as compared to those positive for binding FRAAs [*F*_(1, 92)_ = 9.06, *p* = 0.003 and *F*_(1, 92)_ = 6.76, *p* = 0.01, respectively].

CT was significantly lower in participants positive for the blocking FRAA as compared to those negative for the blocking FRAA [*F*_(1, 92)_ = 4.83, *p* = 0.03] but CT was not significantly different between those positive for blocking FRAA vs those positive for binding FRAAs.

Since supplemental B_12_ can improve the GSH/GSSG ratio in clinical studies, we reanalyzed the difference in GSH/GSSG ratio across groups with B_12_ as a covariate but the results did not changed, indicating that the difference in GSH/GSSG ratio across groups was not due to differences in serum B_12_ concentrations.

The SAM/SAH methylation ratio was not significantly different between blocking FRAA positive and negative groups. Additionally, participants positive for the binding FRAA were not, as a group, significantly different than those negative for the binding FRAA with respect to GSH/GSSG or SAH/SAH ratio or CT.

### Behavior and cognition

The VABS Adaptive Behavior Composite was not significantly different in those positive for the blocking FRAA vs. those negative for the blocking FRAA or those positive for the binding FRAA vs. those negative for the binding FRAA, suggesting that any behavioral differences found across FRAA status were not due to developmental level.

**Figure 3:**
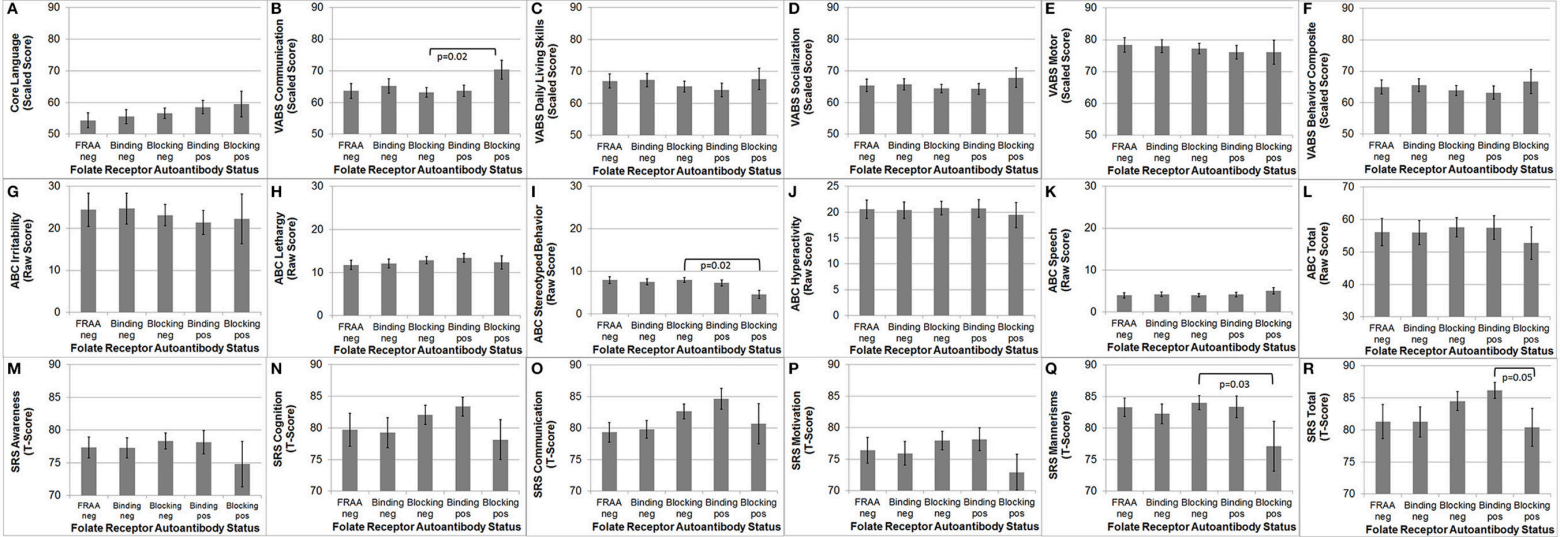
**Children with Autism Spectrum Disorder who are positive for the blocking Folate Receptor Alpha Autoantibody have more favorable (B) communication on the Vineland Adaptive Behavior Scale, (I) Stereotyped behavior on the Aberrant Behavior Checklist (ABC) and (Q) Mannerisms on the Social Responsiveness Scale (SRS) as compared to children negative for the blocking Folate Receptor Alpha Autoantibody**. **(R)** In addition, children positive for the blocking FRAA have better total Social Responsiveness Scale score than those positive for the binding Folate Receptor Alpha Autoantibody. This figure depicts behavioral and Cognitive differences across Folate Receptor Alpha Autoantibody (FRAA) groups including difference in **(A)** Language, **(B–F)** Vineland Adaptive Behavioral Scale, **(G–L)** ABC, and **(M–R)** SRS.

As depicted in Figure 3, VABS Communication [*F*_(1, 86)_ = 5.95, *p* = 0.02], ABC Stereotyped Behavior [*F*_(1, 84)_ = 5.30, *p* = 0.02] and SRS Mannerisms [*F*_(1, 82)_ = 5.19, *p* = 0.03] were significantly better in participants positive for blocking FRAA as compared to those negative for blocking FRAA. Total SRS Score was significantly better in those positive for the blocking FRAA as compared to those positive for the binding FRAA [*F*_(1, 82)_ = 4.04, *p* = 0.05]. None of the behavioral or developmental measures were different across those positive vs. negative for the binding FRAA.

## Discussion

FRAAs (both blocking and binding) are believed to have pathologic consequences in ASD because of their role in blocking the transport of folate into the brain by interfering with FRα function and reducing folate availability in the brain (Frye et al., 2013b). Several studies have documented that individuals with FRAAs, including those with CFD, have ASD characteristics (Ramaekers and Blau, 2004; Ramaekers et al., 2005) and the prevalence of FRAAs appears to be high in individuals with ASD (Frye et al., 2013b; Ramaekers et al., 2013b). However, until this study no one has examined whether individuals with ASD who are FRAA positive have behavioral, developmental and/or physiological characteristics that are distinct from individuals with ASD who are negative for FRAAs.

In this study we found that individuals with ASD who were positive for the blocking FRAA demonstrated behavioral and physiological differences as compared to those negative for the blocking FRAA and as compared to those positive for the binding FRAA. Interestingly this subset of children appears to have better physiological and behavioral profiles, although their behavioral scores are well within the range for children with ASD. There is evidence that children positive for the FRAAs respond to folinic acid therapy, suggesting that these children are a subset of children with ASD that may be particularly responsive to therapy that addresses physiological abnormalities. The fact that children with FRAAs, at least blocking FRAAs, may have less severe ASD symptoms, suggests that these children may be a group to target early as they may be very likely to substantially improve their ASD symptoms and attain optimal outcomes with treatment. This may explain several reports of children with CFD recovering from ASD symptoms with high-dose folinic acid therapy—since they may have started out with milder ASD symptoms it may have been easier for them to recover.

Interestingly, ASD children with the blocking FRAA appear to have a more favorable redox and inflammation profile with relatively better glutathione and CT indices than FRAA blocking negative children. A group of children with ASD and relatively unfavorable redox profiles have been described as having a distinct metabolic endophenotype in previous studies (James et al., 2006). It would appear that the group of ASD children with blocking FRAAs may be a complimentary metabolic endophenotype to the group with unfavorable redox metabolism that may have abnormalities specific to folate metabolism rather than redox metabolism. Importantly, this study demonstrates how multiple biomarkers can be used to differentiate subgroups of children with ASD who could potentially respond to different and/or synergistic treatments.

Children with ASD who are positive for the binding FRAA did not appear to have significant differences in behavioral, developmental or physiological measures when compared to children with ASD who were negative for the binding FRAA but the group did demonstrate significantly higher serum B12 concentrations. This may suggest that the binding FRAAs may interfere with cellular B12 uptake, resulting in a higher concentration of B12 in the blood. Indeed, polymorphisms in the B12 binding protein have been linked to an increased risk of ASD (James et al., 2006). Thus, the binding FRAAs may yet be another mechanism for interfering with B12 metabolism, resulting in methylation and glutathione abnormalities. Abnormalities in B12 metabolism, especially interference with B12 uptake into the cell, could result in the glutathione abnormalities seen in participants with the binding FRAAs. As B12 supplementation can correct glutathione metabolism abnormalities ASD (James et al., 2009) and improve adaptive behavior (Frye et al., 2013a) in children with ASD, it is very possible that a deficit in the cellular uptake of B12, either through a polymorphism in the B12 binding protein or binding FRAA interfering with B12 transport, could result in abnormal glutathione metabolism. Although, we used B12 as a covariate in the analysis of glutathione differences across groups, the covariate would only have been significant if it represented higher levels of intracellular B12. If indeed the higher B12 levels represent a failure of B12 entering the cell, then the covariate would have no relation to glutathione levels (or may have had the opposite predicted relationship). Thus, this is another indication that the elevated B12 levels associated with the binding FRAA do not represent intracellular levels and may be a reflection of a lack of cellular B12 uptake. If this is the case, the binding FRAA may interfere with both folate and B12 transportation, resulting in a more severe phenotype. Indeed, individuals positive for the binding FRAA appear to have worse social skills (as measured by the SRS) than children positive for the blocking FRAA.

Because of the limited sample size we might not have been able to detect small differences in physiology and behavior between the groups. Larger sample sizes could be helpful in detecting more subtle physiological and behavioral differences between groups. Perhaps an interesting study would be to measure the variation in FRAA titers within individuals. Titers tend to vary over short intervals of time (Ramaekers et al., 2013a), but this study only characterized the participants as FRAA positive or negative. It may be very useful to monitor changes in both FRAA titers and behavior within an individual over time as a more sensitive measure of the relationship between FRAAs titers and behavior, as examining intra-subject variation can control for the variation in physiology between individuals. Indeed, one study has demonstrated that the close relationship between aggression and blocking FRAA titers over a 6 week period in a 8 year old girl with ASD (Ramaekers et al., 2007a). Although, this study did not find any relationship between aggressive behavior and FRAA status, this may have been due to the large number of factors that can result in aggressive behavior within an individual with ASD (Matson and Jang, 2014). Thus, intra-subject studies may be most sensitive, especially if considering correlating FRAA titers.

It is important to appreciate that FRAAs could also work in concert with the defects in folate metabolism that have been associated with ASD, including polymorphisms in dihydrofolate reductase (Adams et al., 2007), the reduced folate carrier (James et al., 2006) and methylenetetrahydrofolate reductase (Frustaci et al., 2012), and mitochondrial dysfunction which appears to be rather common in children with ASD (Frye and Rossignol, 2011; Rossignol and Frye, 2012a). In addition, emerging evidence suggests that the enteric microbiome also has an important role for regulating the bioavailability of vitamins such as folate and B12 (Frye et al., 2015). Thus, a study which comprehensively assesses multiple folate pathway abnormalities in relationship to ASD behavior could be very insightful.

This study has helped define the importance of FRAAs in ASD and the effect of the FRAAs on physiology and behavior. It appears that blocking and binding FRAAs are associated with slightly different physiological and behavioral effects in children with ASD. Further, research will help better define their physiological roles and significance. Studying clinical subgroups in other disorders associated with FRAAs such as schizophrenia (Ramaekers et al., 2014) and subfertility (Berrocal-Zaragoza et al., 2009) may also yield important information. Ultimately, this information may be important to determining optimal treatments for certain children with ASD.

## Author contributions

RF designed the study, analyzed the data and wrote the manuscript; LD collected and analyzed the data and edited the manuscript; JS collected and analyzed the data and designed the study and edited the manuscript; MT collected the data and edited the manuscript; RW collected and analyzed the data; SR collected and analyzed the data and edited the manuscript; SK edited the manuscript; SB analyzed the data; SM collected and analyzed the data and designed the study and edited the manuscript; JS collected and analyzed the data; EQ designed the study and wrote the manuscript.

### Conflict of interest statement

The authors declare that the research was conducted in the absence of any commercial or financial relationships that could be construed as a potential conflict of interest. RF is on the scientific advisory board of Iliad Neurosciences, Inc (Plymouth Meeting, PA). Two of the authors (JS and EQ) are inventors on a US patent for the detection of FR autoantibodies issued to the Research Foundation of the State University of New York.
